# *Aspergillus flavus* Conidia-derived Carbon/Sulfur Composite as a Cathode Material for High Performance Lithium–Sulfur Battery

**DOI:** 10.1038/srep18739

**Published:** 2016-01-06

**Authors:** Maowen Xu, Min Jia, Cuiping Mao, Sangui Liu, Shujuan Bao, Jian Jiang, Yang Liu, Zhisong Lu

**Affiliations:** 1 Institute for Clean Energy & Advanced Materials, Faculty of Materials and Energy, Southwest University, Chongqing 400715, P.R. China; 2 Chongqing Key Laboratory for Advanced Materials and Technologies of Clean, Energies, Chongqing 400715, P.R. China; 3 Institute of Agro-Products Processing Science and Technology, Chinese Academy of Agricultural Sciences/Key Laboratory of Agro-Products Processing, Ministry of Agriculture, Beijing 100193, P.R. China

## Abstract

A novel approach was developed to prepare porous carbon materials with an extremely high surface area of 2459.6 m^2^g^−1^ by using *Aspergillus flavus* conidia as precursors. The porous carbon serves as a superior cathode material to anchor sulfur due to its uniform and tortuous morphology, enabling high capacity and good cycle lifetime in lithium sulfur-batteries. Under a current rate of 0.2 C, the carbon-sulfur composites with 56.7 wt% sulfur loading deliver an initial capacity of 1625 mAh g^−1^, which is almost equal to the theoretical capacity of sulfur. The good performance may be ascribed to excellent electronic networks constructed by the high-surface-area carbon species. Moreover, the semi-closed architecture of derived carbons can effectively retard the polysulfides dissolution during charge/discharge, resulting in a capacity of 940 mAh g^−1^ after 120 charge/discharge cycles.

Although lithium-ion (Li-ion) batteries with high energy densities have dominated power sources of portable electronic devices, they fail to meet requirements in large-scale applications of electrical vehicles and grids because of cost, safety and capacity limitations from intercalating-type cathode materials such as LiCoO_2_, LiFePO_4_ and Li_2_MnO_4_^1^,^2^,^3^. Researchers in battery communities aim to develop high capacity cathodes to replace the current interaction compounds. Under this circumstance, Li-air and Li-S batteries have been thus proposed and paid tremendous attention in recent years as promising alternatives to conventional Li-ion batteries^4^,^5^,^6^.

As a cathode material for Li-S battery, sulfur can theoretically deliver a large specific capacity of 1675 mAh g^−1^ and a specific energy up to 2600 Wh kg^−1^, almost ten times over those of intercalation compounds^7^. Moreover, the use of sulfur could remarkably reduce the battery cost since it is one of the most abundant elements in the earth^8^. Nevertheless, the low conductivity and poor cycling performance of sulfur greatly restrict its use in Li-S batteries^9^,^10^. To overcome the barriers, sulfur has been incorporated into various carbon materials such as microporous carbon^11^,^12^, carbon nanofiber^13^,^14^,^15^,^16^, carbon nanotubes^17^,^18^,^19^,^20^ and graphene^21^,^22^,^23^,^24^ to form functionalized carbon/sulfur composites for battery applications. Among them, porous carbons may act as good host matrices and help to build an electrically conductive network for sulfur cathode^25^,^26^. The pioneering work was reported by Nazar *et al.*^27^ to directly encapsulate sulfur in mesoporous carbon particles, demonstrating a relatively high capacity of about 1000 mAh g^−1^ for 20 cycles. The porous structures can effectively seize sulfur in carbonaceous matrices and prevent it from dissolving into the electrolyte during charge/discharge, improving the cycle life of Li-S battery^28^. Various approaches have been developed to synthesize carbon materials with porous structures recently. Zhao *et al.*^29^ developed a solvent-evaporation-induced coating and self-assembly way to obtain porous carbons, well confining the sulfur within the cathode region. Oschatz *et al.*^30^ achieve a unique network-like carbon hosts from poly(methyl methacrylate) (PMMA) spheres. In addition, porous carbons made from metal organic frameworks (MOFs), as reported by Xia *et al.*, were also confirmed to be good host matrix for sulfur^31^. Based on extensive efforts, the performance of Li-S batteries has been improved significantly in the past decade. However, these porous carbon materials aforementioned are still facing formidable challenges arising from their high prices and complicated synthesis procedures.

Beyond conventional methods with inorganic precursors, we herein present a unique biological approach, via using Aspergillus *flavus* (A. *flavus*) conidia for the first time, to prepare porous carbon materials with an extremely high surface area of 2459.6 m^2^g^−1^. The as-prepared carbon/sulfur composite demonstrates a high reversible capacity, good rate capability and long-term cycling stability, showing great potential in future applications of Li-S batteries.

## Results

### Synthesis and characterization

**Figure 1:**
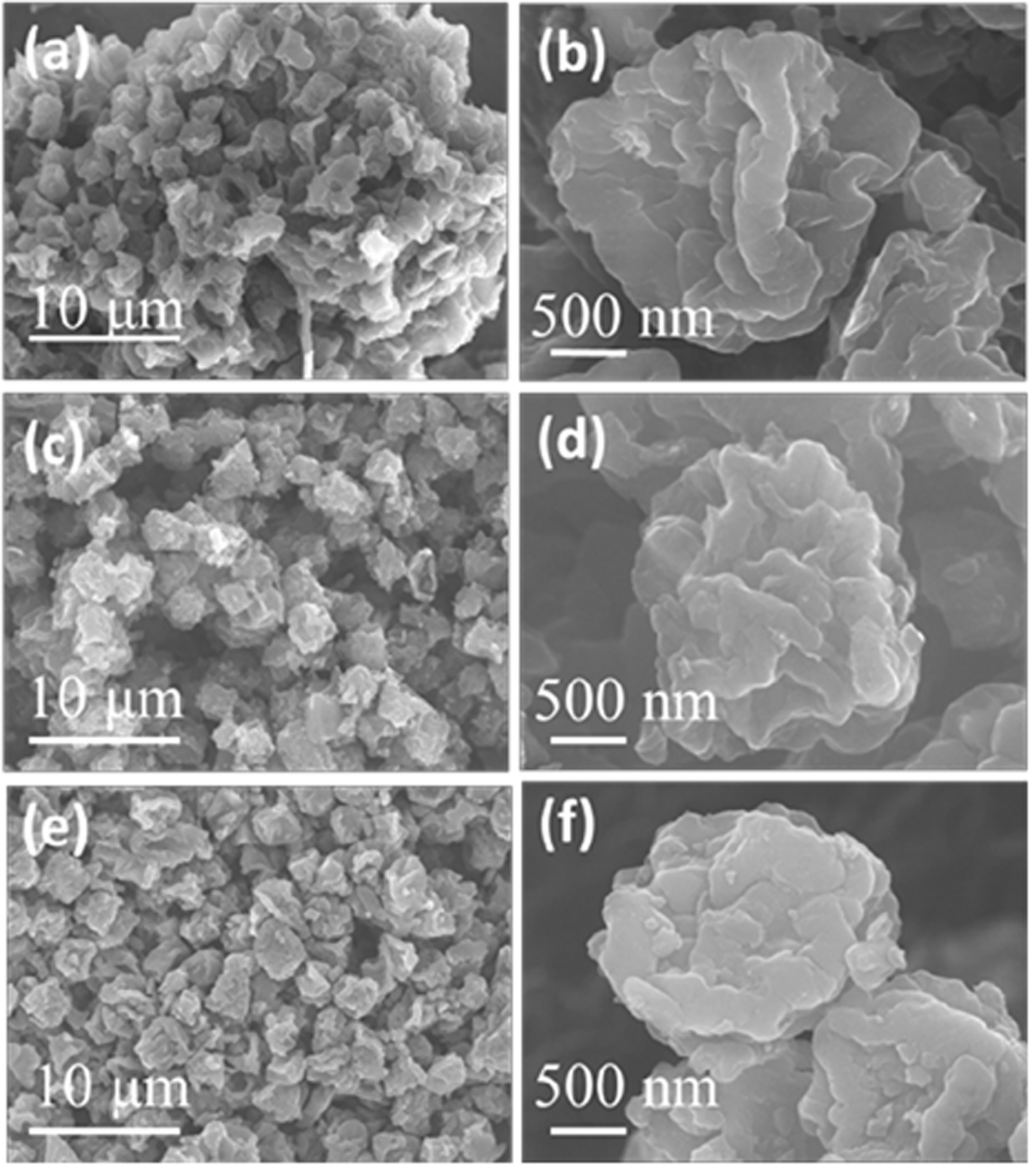
SEM images of (a),(b) CM; (c),(d) PCM; (e),(f) PCM/S.

Geometric morphologies of CM, PCM and PCM/S were initially observed by SEM. Figure 1(a) shows a representative SEM overview of CM samples, which are evidently made of aggregated microspheres. The CM spheres with an average diameter of ~1.5 μm possess plicate and rough surfaces covered by plenty of wrinkles and folds (Fig. 1b). Clearly, after KOH activation, the plicate spheres become relatively scattered when compared to the case of CM (Fig. 1c,d)). The SEM images of PCM/S composites were displayed in Fig. 1(e,f). Distinctly from the pristine PCM, the PCM/S composites become more hardened. In comparison to the PCM, the spheres have a relatively smoother surface, which may be due to the fusion of sulfur into PCM.

**Figure 2:**
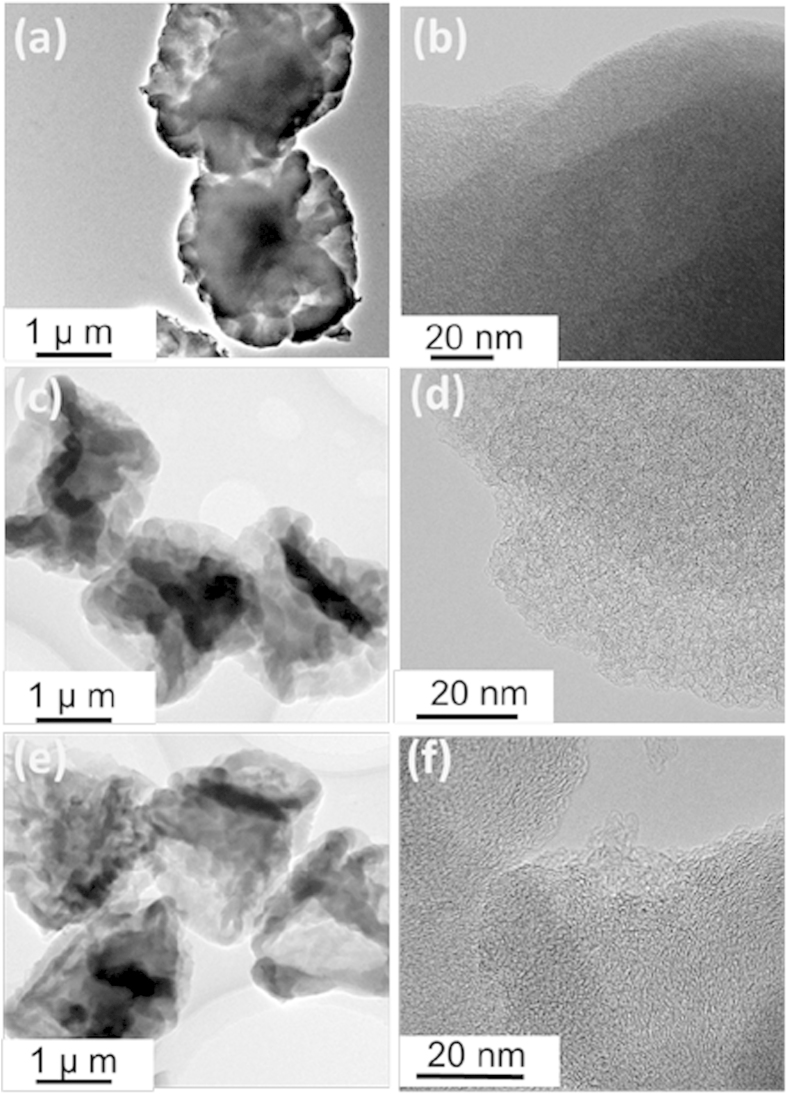
TEM images of (a),(b) CM; (c),(d) PCM; (e),(f) PCM/S.

**Figure 3:**
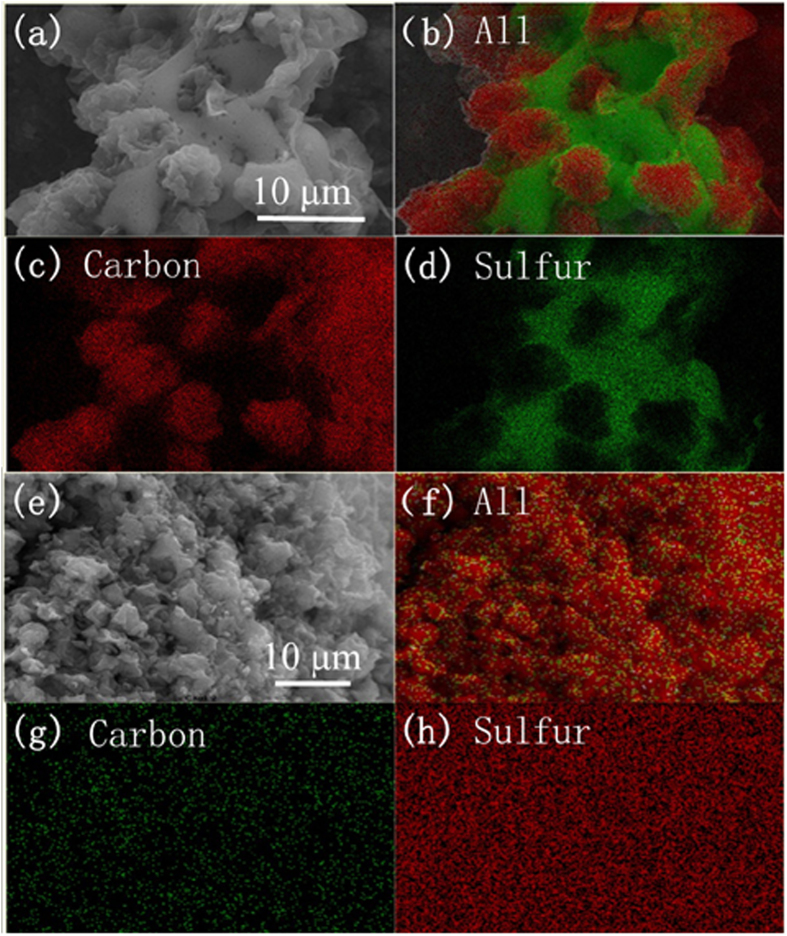
(**a**) SEM image and (**b–d**) EDS mappings of the CM/S composite sample (**e**) SEM image and (**f–h**), EDS mappings of PCM/S composite sample.

The CM, PCM and PCM/S samples were further characterized by TEM. As shown in Fig. 2a, the CM well inherits the plicate texture of *A. flavus* conidia. After carbonized at 600 °C, highly disordered macropores can be observed in CM (Fig. 2a,b). After undergoing KOH activation, the surface of PCM becomes much slacker and rougher (Fig. 2c). The magnified TEM image (Fig. 2d) reveals that a large number of pores are homogeneously dispersed on PCM. Morphologies and microstructures of PCM/S composites were presented in Fig. 2e,f, signifying that their spherical profiles could still be remained. However, the PCM/S composites become even dense. Particularly, some fingerprint-liked structures are presented (Fig. 2f), which may be due to the successful fusion of sulfur into the PCM pores. The SEM-EDS elemental mappings were further used to characterize the sulfur distribution in the carbon materials. Obvious boundary lines of elemental C and S can be well distinguished in Fig. 3a–d, indicating that sulfur cannot enter into CM. Sulfur particles are actually covering on the spherical samples. The findings are highly in agreement with the TEM results. Figure 3e–h successively show the SEM observation and EDS elemental mapping records toward PCM/S. Clearly, sulfur has been homogeneously embedded into the PCM reservoirs.

**Figure 4:**
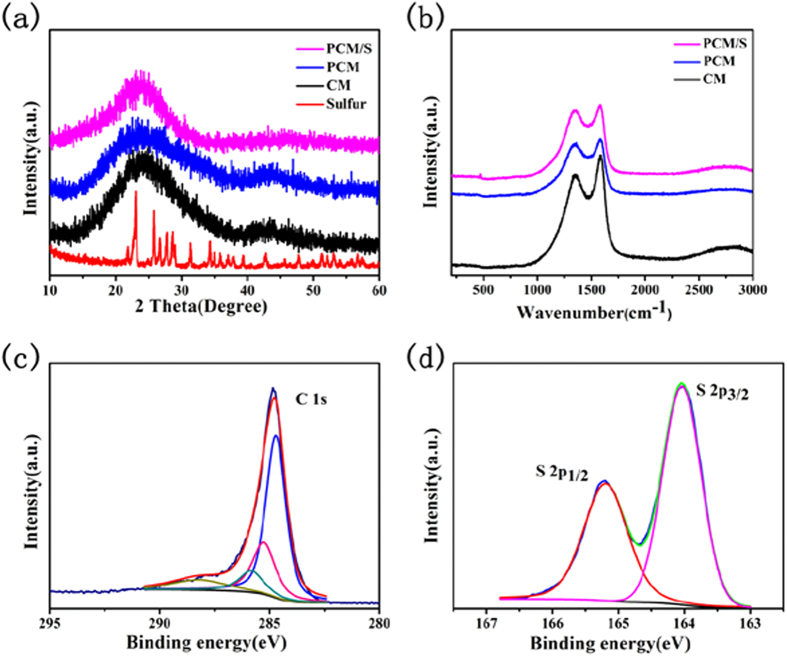
(**a**) XRD profiles of CM (black), PCM (blue), PCM/S (purple) and sulfur(red); (**b**) Raman spectra of CM (black), PCM (blue) and PCM/S (purple); XPS of carbon (**c**) and S (**d**) of the PCM/S composite.

**Figure 5:**
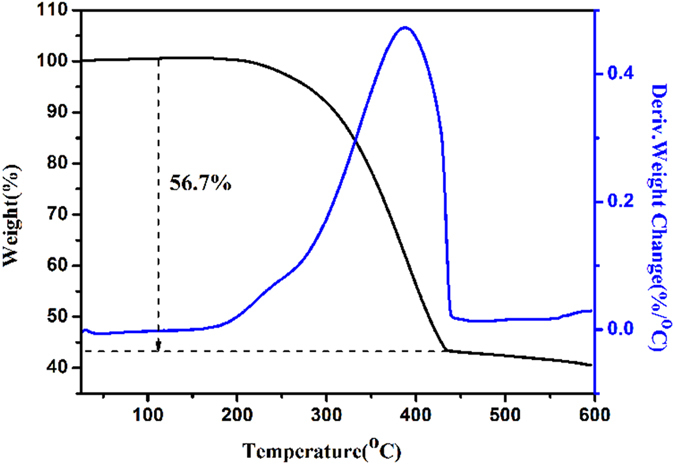
TG curves of the PCM/S under N_2_ at a heating rate of 10 °C min^−1^.

The X-ray diffraction (XRD) patterns of sulfur (red), CM (black), PCM (blue) and PCM/S (purple) samples are presented in Fig. 4a. Two broad peaks are observed in XRD patterns of CM and PCM materials. The one located at around 24^o^ may be attributed to (002) reflection of graphitic planes. The peak at around 44^o^ may be due to the superposition of (100) and (101) reflections of the graphitic-type lattices. The characteristic peaks of sulfur cannot be detected for PCM/S composites, suggesting that sulfur has successfully diffused into pore structures of PCM and been highly dispersed^30^. Raman patterns of CM (black), PCM (blue) and PCM/S (purple) composites are shown in Fig. 4b. Two peaks lying at about 1330 cm^−1^ and 1590 cm^−1^, which are respectively well known as D-band and G-band of carbon, can be found in the spectra of all samples. Generally, the D band is ascribed to the disordered graphite structure whereas the G band is denoted as the presence of crystalline graphitic carbon^32^. The intensity ratio (I_D_/I_G_) can be used to estimate the defective degree of carbon materials^7^. The I_D_/I_G_ of CM, PCM, PCM/S are calculated to be 0.82, 0.95 and 0.97, respectively. The I_D_/I_G_ enhancement of PCM may be attributed to the defects and highly porous structures that are created during KOH treatment^33^, agreeing well with the TEM observations. The I_D_/I_G_ ratio of PCM/S suggests that more lattice defects are generated, which may arise from the merging of sulfur into the PCM lattice. The XPS spectra of C 1s and S 2p of PCM/S are displayed in Fig. 4(c,d). The C1s peak can be well fitted with four isolated peaks. The peak above 286 eV is assigned to the surface of amorphous carbon and the lowest environment carbon, while the one lying at around 284.5 eV indicates the existence of graphitic-type carbon, in accordance with the profile of our XRD analysis. The peak at around 285.4 eV could be attributed to the presence of sulfur-bound or aliphatic-like carbon species^34^. Figure 4d shows the appearance of S 2p_1/2_ and S 2p_3/2_ peaks. The position of S 2p_3/2_ peak is measured at about 163.8 eV, indicating the existence of sulfur-doped carbonaceous materials wherein an aromatic C−S−C type bonding is involved^35^. Combined with C 1s peak location aforementioned, it is highly suggested that the C-S bond could indeed exist in our PCM/S composite. Thermogravimetric analysis (TGA) toward PCM/S composite was carried out under N_2_ atmosphere (Fig. 5) so as to confirm the content of sulfur. There is only one weight loss stage observed in TGA curve. In the temperature range from 190 °C to 430 °C, the mass loss of sulfur in the PCM/S composite is determined to be ~56.7 wt%. From Fig. S1, the mass loss of ~59.7 wt% from 180 °C to 290 °C reveals the actual content of sulfur in CM/S composite.

**Figure 6:**
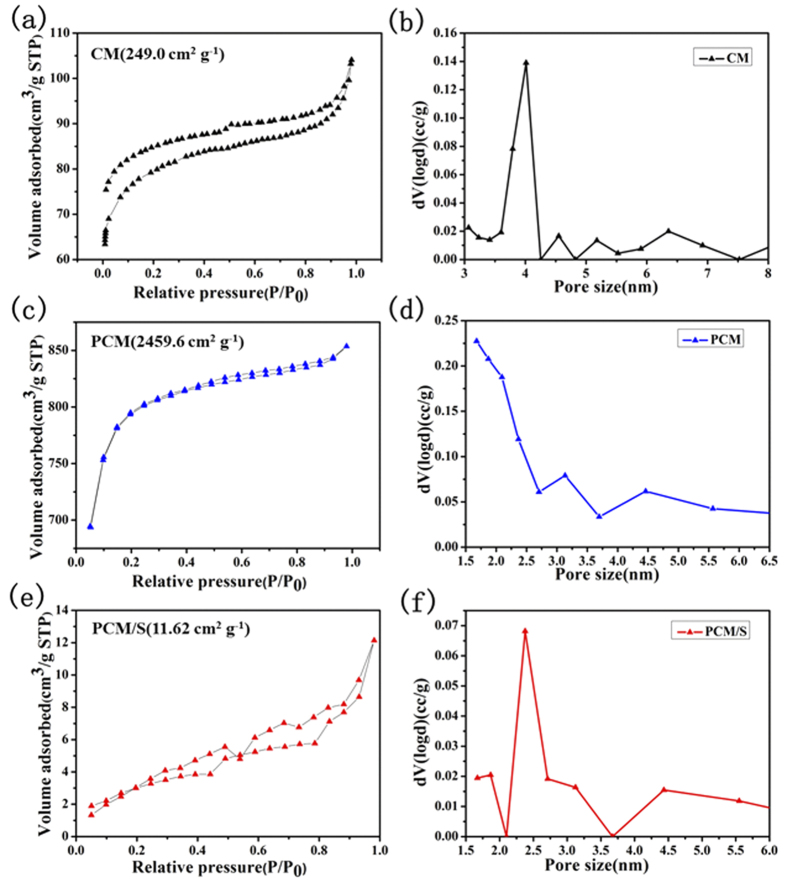
N_2_ adsorption-desorption isotherms and the pore size distributions for distinct samples: (a and b) CM sample; (c and d) PCM; (e and f) PCM/S composite.

The BET surface area of PCM is measured to be 2459.6 m^2^g^−1^ (Fig. 6c), which is almost 10 times more than that of CM (249.0 m^2^g^−1^, see Fig. 6a). The pore size distribution of PCM (Fig. 6d) indicates that the material has a well-defined mesoporous structure with a central pore size ranging from 1.5 to 3.5 nm. In order to further prove the immersion of sulfur into porous carbon materials, the specific surface area of the PCM/S composite was also tested (Fig. 6e). The specific surface area of PCM/S composite reduced sharply from 2459.6 to 11.62 m^2^g^−1^ (Table 1). This implies that the sulfur was infiltrated into the nanoporous of the PCM, and is fully consistent with our TEM and EDS results.

### Electrochemical Performance

**Figure 7:**
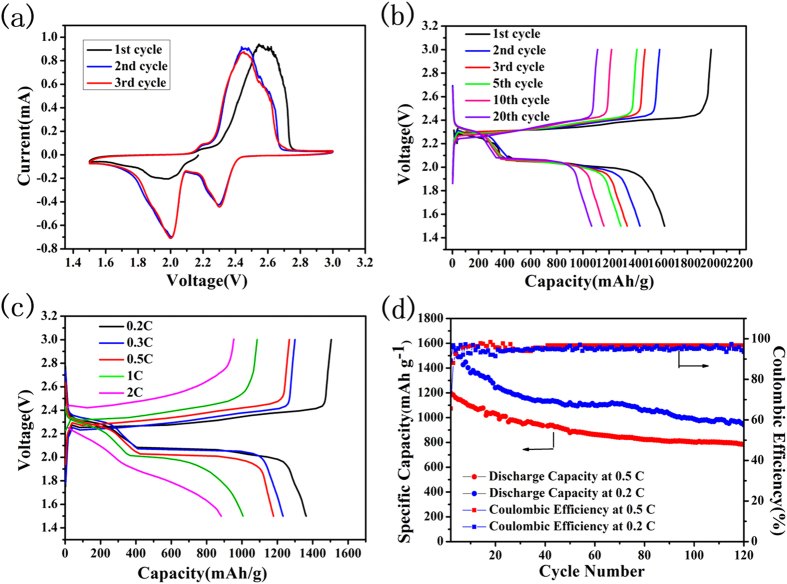
(**a**) CV curves of the PCM/S cathode in a coin cell at a scan rate of 0.1 mV s^−1^. (**b**) Charge-discharge profiles of PCM/S at different cycle numbers at a rate of 0.2 C (1C = 1675 mA g^−1^). (**c**) Rate performance of the PCM/S composite cathode under different current rates. (**d**) Cycling performance and Coulombic efficiency at the discharge rate of 0.2 C and 0.5 C.

The CV of the PCM/S composite electrode is shown in Fig. 7a, with a scanning rate of 0.1 mV s ^−1^ from the 1^st^ to 3^rd^ cycle. Two main reduction peaks at 2.3 V and 2.05 V are observed, respectively, which are tightly associated with the interactions between Li and sulfur according to energy-storage mechanisms in lithium-sulfur batteries^8^. Within a two-step discharging process, sulfur is firstly reduced to S^8-^ (in accordance with the reduction peak at 2.3 V), and furthermore changed into S^2-^, as related to a reduction peak emerging at 2.05 V^36^,^37^. Figure 7b shows the charge-discharge profile of PCM/S composite under a current density of 0.2 C (1C = 1675 mA g^−1^) at the 1^st^, 2^nd^, 3^rd^, 5^th^, 10^th^ and 20^th^ cycle, respectively. The PCM/S exhibits excellent performance with an initial specific capacity of 1625 mAh g^−1^. This may be attributed to the good electrons conductivity of the overall hybrid system. After 20 cycles, the discharge capacity is close to 1070 mAh g^−1^. Also, two obvious discharge plateaus could be found in this curve, which is consistent with CV peaks described above. The rate performance of the PCM/S composite electrode is summarized in Fig. 7c. The cell was operated at five different current densities of 0.2 C, 0.3 C, 0.5 C, 1 C and 2 C in a voltage window of 1.5 ~ 3 V, respectively. As expected, PCM/S demonstrates the favorable rate performance. It achieves a discharge capacity of ~1250 mAh g^−1^; even at a large current density of 2 C, the discharge capacity can still reach ~900 mAh g^−1^. In addition to its considerable initial capacity, the PCM/S electrode also shows stable cyclic performance (Fig. 7d). The discharge capacity can be achieved up to nearly 800 mAh g^−1^ after 120 cycles under a current density of 0.5 C. Also for the lower current discharge test at 0.2 C, the PCM/S composite still retains 940 mAh g^−1^, revealing its good cycling behavior in Li-S batteries. From close observations toward cycled electrodes (Fig. S2), we found that the hybrid cathode can almost remain its original structures even after 120 cycles, unquestionably confirming its good stability in battery applications. In sharp contrast, the discharge capacity of CM/S composite is only ~300 mAh g^−1^ after 120 cycles at a current density of 0.5 C (Fig. S3), which highly suggests the critical role of porous structures in PCM/S electrode.

## Discussion

A novel method was developed to fabricate PCM with extremely large surface areas up to 2459.6 m^2^g^−1^ by using *A. flavus* conidia as precursors. The experimental results suggest that the PCM material is a reliable host matrix for sulfur reservoirs because of its high surface area and rich pore structures. The PCM/S electrodes with 56.7 wt% sulfur loading exhibit higher reversible capacity, better rate capability and longer cycling lifetime in comparison to the CM/S counterparts. This may be due to a fact that PCM with adequate small pores can function as a good sulfur host, improving the sulfur utilization rate and well restraining the dissolution of polysulfides into electrolytes.

## Methods

### Materials preparation

*A. flavus* conidia were initially washed by glutaraldehyde and alcohol, and then dried at room temperature for 24 h. The dried conidia were carbonized at 600 °C for 2 h with a heating rate of 2 °C min^−1^. The obtained carbon material from *A. flavus* conidia was called CM for short. To fabricate porous carbon material (PCM), the CM was grinded with KOH (w_c_:w_KOH_ = 1:2) for 15 min. The mixtures were subsequently calcined at 300 °C for 1.5 h and at 750 °C for 2 h with a heating rate of 5 °C min^−1^ in a quartz-tube furnace under Ar atmosphere. The samples were washed with 1 M HCl and deionized water for three times, followed by drying at 80 °C for 12 h in a vacuum oven.

The combination of CM or PCM with sulfur was prepared by a simple melting diffusion way. The as-prepared CM and the sublimed S were grinded together with a mass ratio of w_c_:w_s_ = 2:3. The mixture was then heated in a tubular furnace at 155 °C for 10 h at a heating rate of 3 °C min^−1^ under Ar atmosphere. The as-formed hybrids were called CM/S and PCM/S, respectively.

### Material characterization

Crystal structures of the samples were characterized by X-ray diffractometer (Maxima-X XRD-7000) and Cu K-alpha radiation (λ = 1.5406 nm) over the 2θ range of 10°–60°. Morphology and microstructures of the as-prepared products were examined by field-emission scanning electron microscopy (FESEM, JSM-7800N) and transmission electron microscopy (TEM, JEM-2100). Raman spectra were obtained using a HORIBA Scientific LabRAM HR Raman spectrometer system equipped with a 532.4 nm laser as the exciting radiation. The weight percent of sulfur was determined by thermogravimetric analyzer (TGA, Q50). X-ray photoelectron spectroscopy (XPS) measurements were performed on a Thermo Scientific ESCALAB 250Xi electron spectrometer. Nitrogen adsorption−desorption isotherms and pore size distribution were characterized by Quadrasorb evo 2QDS-MP-30 (Quantachrome Instruments, USA).

### Electrochemical measurements

The electrodes were fabricated by coating a slurry (75% active material, 15% carbon black and 10% polyvinylidenefluoride (PVDF) binder dissolved in N-methyl pyrolidine (NMP)) onto an aluminum foil and then dried in vacuum at 55 °C for 15 h. CR3025 coin cells were used and assembled in an argon-filled glove box. Li foil was utilized as the counter electrode. The electrolyte was 1 M bis(trifluoromethane) sulfonimide lithium salt (LiTFSI) dissolved in a mixture of 1,3-dioxolane (DOL) and dimethoxymethane (DME) with a volume ratio of 1:1, with 0.1 M LiNO_3_ as the electrolyte additive. All cells were aged for several hours before charge–discharge to ensure the adequate penetration of electrolyte into the electrode. The cells were galvanostatically charged and discharged between 1.5 V and 3 V (versus Li/Li^+^) using a Land instruments testing system.

## Additional Information

**How to cite this article**: Xu, M. *et al.* *Aspergillus flavus* Conidia-derived Carbon/Sulfur Composite as a Cathode Material for High Performance Lithium–Sulfur Battery. *Sci. Rep.* **6**, 18739; doi: 10.1038/srep18739 (2016).

## Supplementary Material

Supplementary Information

## Figures and Tables

**Table 1:** Porosity properties for CM, PCM and PCM/S.

sample name	BET area (m^2^ g^−1^)	pore volume (cc g^−1^)	Pore size (nm)
CM	249.0	0.16	3~4.3
PCM	2459.6	1.30	1.5~3.5
PCM/S	11.62	0.019	2~3.5
