# Supplementary material for: Aspergillus flavus Conidia-derived Carbon/Sulfur Composite as a Cathode Material for High Performance Lithium–Sulfur Battery
Source: Sci Rep. 2016 Jan 6;6:18739. doi: 10.1038/srep18739 (PMC4702123; doi:10.1038/srep18739)
Supplement: Supplementary Information [file srep18739-s1.pdf]

Supporting information:

Aspergillus flavus Conidia-derived Carbon/Sulfur Composite as a Cathode Material for High Performance Lithium−Sulfur Battery

Maowen Xu[1,2,†,*], Min Jia[1,2, †], Cuiping Mao[1,2], Sangui Liu[1,2], Shujuan Bao[1,2], Jian Jiang[1,2], Yang Liu[3] and Zhisong Lu[1,2,*]

Affiliation:

[1]Institute for Clean Energy & Advanced Materials, Faculty of Materials and Energy, Southwest University, Chongqing 400715, P.R. China

[2]Chongqing Key Laboratory for Advanced Materials and Technologies of Clean Energies, Chongqing 400715, P.R. China

[3] Institute of Agro-Products Processing Science and Technology, Chinese Academy of Agricultural Sciences/Key Laboratory of Agro-Products Processing, Ministry of Agriculture, Beijing 100193, P. R. China

Corresponding authors*
E-mail: E-mail:xumaowen@swu.edu.cn; zslu@swu.edu.cn

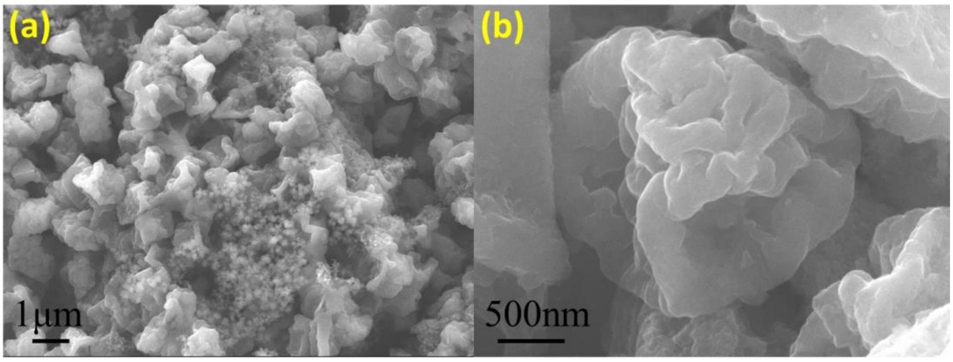

Fig. S1 SEM images of cathode composite after 120 cycles.

From the different magnification of the SEM images, we can see that, after 120 cycles, the cathode composite mainly remain its primary feature, indicating that the structure of the composite is stable.

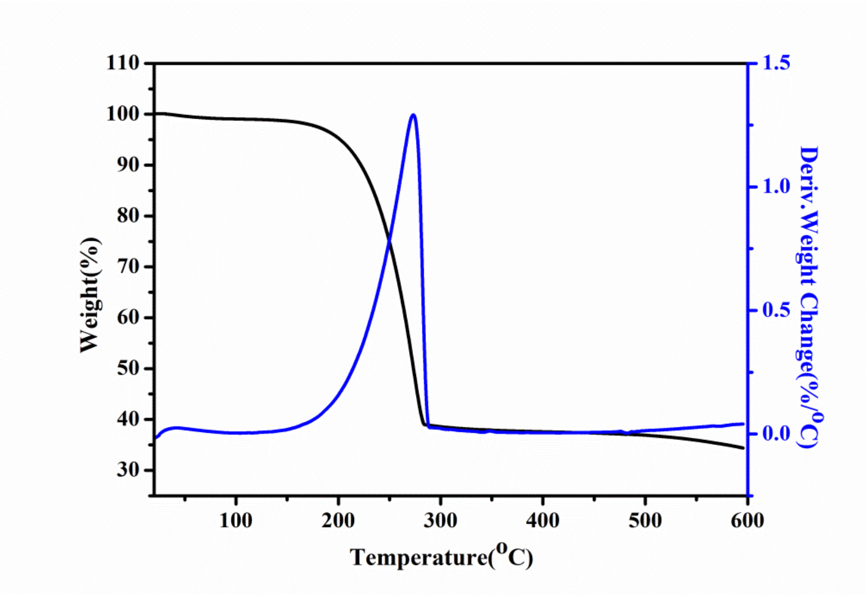

Fig. S2 TG curves of CM/S under N2 atmosphere at the heating rate of 10 ℃ min^{-1} from 20 ℃ ~ 600 ℃.

Thermogravimetric analyses of the CM/S composite was carried out under N_2 atmosphere. From 180 ℃ to 290 ℃, the mass loss of CM/S composite is 59.7wt%, which is the content of sulfur.

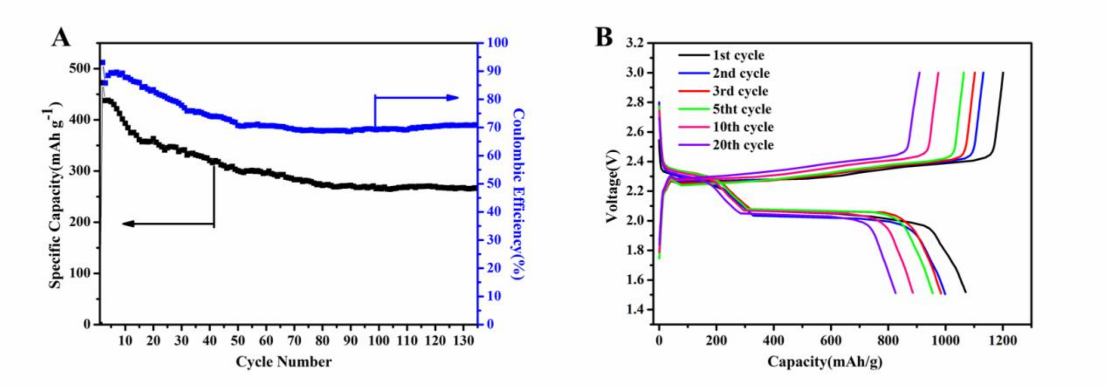

Fig. S3 (A) Cycling performance and coulombic efficiency at a discharge rate of 0.5 C, and (B) Charge-Discharge profiles of CM/S of different cycle at a rate of 0.2 C of CM/S composite.

The discharge capacity is only about 300 mAh g^{-1} after 135 cycles at a current density of 0.5 C (Fig. S2A). Fig. S2B shows charge-discharge profiles of 1st, 2nd, 3rd, 5th, 10th, 20th cycle of CM/S composite at the current density 0.2 C. The CM/S reveals the initial capacity of 1070 mAh g^{-1}. But after 20 cycles, the discharge capacity is close to 820 mAh g^{-1}.
